# Mitochondrial Integrity and Kynurenine Pathway Enzyme Dynamics in the Hippocampus of Rats with Scopolamine-Induced Cognitive Deficits

**DOI:** 10.3390/ijms26209883

**Published:** 2025-10-11

**Authors:** Mariola Herbet, Angelika Tkaczyk-Wlizło, Katarzyna Wicha-Komsta, Bartosz Twarowski, Brygida Ślaska, Tomasz Kocki, Krzysztof Kowal, Iwona Piątkowska-Chmiel

**Affiliations:** 1 Chair and Department of Toxicology, Faculty of Pharmacy, Medical University of Lublin, Jaczewskiego 8b Street, 20-090 Lublin, Poland; bartosz.twarowski@interia.pl (B.T.); iwona.piatkowska-chmiel@umlub.edu.pl (I.P.-C.); 2 The Sub-Department of General and Molecular Genetics, Institute of Biological Bases of Animal Production, University of Life Sciences in Lublin, Akademicka 13 Street, 20-950 Lublin, Poland; angelika.tkaczyk@up.lublin.pl (A.T.-W.); brygida.slaska@up.lublin.pl (B.Ś.); krzysztof.kowal@up.lublin.pl (K.K.); 3 Chair and Department of Experimental and Clinical Pharmacology, Faculty of Medicine, Medical University of Lublin, Jaczewskiego 8b Street, 20-090 Lublin, Poland; katarzyna.wicha-komsta@kul.pl (K.W.-K.); tomasz.kocki@kul.pl (T.K.); 4 Institute of Health Sciences, The John Paul II Catholic University of Lublin, Konstantynów 1F Street, 20-708 Lublin, Poland

**Keywords:** cognitive impairment, mitochondrial DNA, D-loop region, KP, hippocampus, scopolamine, neurodegeneration

## Abstract

Cognitive impairments, particularly in the context of neurodegenerative diseases, are associated with disruptions in mitochondrial function and key metabolic pathways. This study investigates the impact of short-term scopolamine exposure on mitochondrial DNA (mtDNA) stability and the kynurenine pathway (KP) in the hippocampus, a brain region central to learning and memory. We analyzed the mitochondrial D-loop region for mutations and heteroplasmy levels in hippocampal tissue from rats exposed to scopolamine (1 mg/kg/0.4 mL/cc *i.p.* x 14 days). Additionally, the expression of the KP enzymes kynurenine aminotransferase (KAT I, KAT II) and kynurenine 3-monooxygenase (KMO) and receptors aryl hydrocarbon receptor (Ahr) and G protein-coupled receptor 35 (GPR35) was evaluated using quantitative PCR. Neither significant mutation nor heteroplasmy changes were observed in the mtDNA D-loop region between the scopolamine-treated and control groups. Similarly, the hippocampal expression levels of the *kat I*, *kat II*, *kmo* and *ahr* and *gpr35* genes remained unchanged, indicating no activation of this metabolic pathway under short-term scopolamine exposure. These findings suggest that the mitochondrial genome in the hippocampus remains stable under acute pharmacological stress induced by scopolamine, with no significant activation of the KP. These results underline the distinction between transient, reversible cognitive deficits and chronic neurodegenerative processes, providing insights for therapeutic approaches targeting specific stages of cognitive change.

## 1. Introduction

Cognitive impairment is a serious health problem, especially in the context of neurodegenerative diseases such as Alzheimer’s disease. These disorders not only impair learning and memory, but also seriously affect the quality of life of those affected. Among the brain areas involved in cognitive functions, the hippocampus plays a key role due to its involvement navigation, learning processes, and memory formations [1]. This structure is highly susceptible to molecular, cellular, and structural changes that can disrupt its functions and contribute to cognitive decline. The hippocampus is characterized by high metabolic demands and complex neuronal circuits, making it susceptible to a variety of insults, including oxidative stress, mitochondrial dysfunction, and synaptic dysregulation [2].

Studies on the pathophysiology of cognitive deficits increasingly rely on animal models to reproduce the complex processes involved in these disorders. Among them, the scopolamine-induced cognitive deficit model is widely used in scientific research. Scopolamine, a nonselective antagonist of muscarinic acetylcholine receptors, effectively disrupts cholinergic neurotransmission, a hallmark of cognitive dysfunction in Alzheimer’s disease and other neurodegenerative disorders [3]. This model mimics the neurochemical disturbances seen in these diseases, providing a valuable tool for understanding the molecular and cellular mechanisms that drive cognitive decline. Administration of scopolamine causes transient changes in cognitive performance, manifest as amnestic-like changes learning and/or memory [4].

Mitochondria are essential for maintaining the high energy demands of neurons. These organelles not only produce adenosine triphosphate (ATP) via oxidative phosphorylation, but also regulate vital cellular processes including calcium homeostasis, reactive oxygen species (ROS) production, and apoptotic signaling [5]. One of the key regions in mitochondrial DNA (mtDNA) is the D-loop, a noncoding segment that plays a key role in regulating mtDNA replication and transcription [6]. The D-loop contains essential promoter regions and binding sites for transcription factors such as mitochondrial transcription factor A (TFAM), which is essential for the maintenance and expression of the mitochondrial genome [6,7]. Due to its regulatory importance and single-stranded structure during replication, the D-loop is highly susceptible to damage, mutations, and heteroplasmic rearrangements. Mutations in this area can disrupt mitochondrial function and lead to impaired energy production, increased oxidative stress, and disruptions in mitochondrial biogenesis, although, as scientists emphasize, their clinical significance requires confirmation in further studies [8,9]. Such alterations have been implicated in the pathophysiology of a wide range neurodegenerative diseases, including Alzheimer’s disease. Specifically, single-nucleotide polymorphisms (SNPs) and heteroplasmic shifts in within the D-loop region of mitochondrial DNA (mtDNA) can impair transcriptional efficiency, diminish mitochondrial respiratory capacity, and promote neuronal apoptosis [10]. The observed accumulation of D-loop damage observed in Alzheimer’s disease may therefore serve as a critical biomarker of mitochondrial dysfunction and oxidative stress, key drivers of neurodegeneration [11].

In recent years, considerable attention has been paid to understanding the KP and its related receptors, including *Ahr* and *GPR35*, in the context of neurochemistry and the pathogenesis of neurodegenerative diseases [12,13]. The KP is a major pathway of tryptophan catabolism leading to the production of several neuroactive metabolites that play a dual role in maintaining neuronal homeostasis and contributing to neurotoxicity under pathological conditions. Key enzymes in this pathway, such as KAT I and KAT II and KMO, play a pivotal role in modulating the balance between neuroprotective and neurotoxic metabolites [14,15]. KAT I and KAT II catalyze the conversion of kynurenine to kynurenic acid (KYNA), a neuroprotective compound that acts as an antagonist of NMDA and α7 nicotinic acetylcholine receptors, thereby modulating excitotoxicity and inflammation. In turn, KMO directs kynurenine metabolism toward the production of 3-hydroxykynurenine and quinolinic acid, metabolites associated with oxidative stress and excitotoxic damage. Dysregulation of these enzymes can lead to an imbalance of KP metabolites, which in turn affects neuroinflammatory processes, synaptic plasticity, and neuronal viability [16]. Elevated levels of quinolinic acid are associated with excitotoxicity and oxidative stress, which are key pathological mechanisms in neurodegenerative diseases such as Alzheimer’s disease [17]. In contrast, altered levels of kynurenic acid have been linked to cognitive deficits and mood disorders, highlighting the complex role of this pathway in brain function [18]. Receptors, such as Ahr and GPR35, also play key roles in this pathway [19]. Ahr, a ligand-activated transcription factor, mediates responses to kynurenine-derived ligands by influencing gene expression programs that regulate inflammation, detoxification, and cellular metabolism. GPR35, a G protein-coupled receptor activated by kynurenic acid, is involved in modulating immune responses and neuronal excitability [20,21]. Both receptors are increasingly recognized as key mediators linking metabolic changes in the KP to broader neurochemical and neurophysiological outcomes. Changes in the expression of Ahr, GPR35, and KP enzymes may modulate the neuronal response to damaging agents, influencing the progression and severity of cognitive impairment. In experimental models of neurodegeneration and cognitive dysfunction, such as scopolamine-induced deficits, investigating these molecular pathways offers valuable insight into mechanisms underlying of neuronal resistance or vulnerability.

We hypothesized that short-term scopolamine exposure induces reversible changes in cognitive performance without permanent effects on the molecular endpoints analyzed. Understanding these molecular changes may provide insight into the mechanisms underlying cognitive performance and help identify potential targets for therapeutic intervention in neurodegenerative conditions. To address this: Adult, male, Wistar rats were randomly assigned to receive vehicle (sterile phosphate-buffered saline with 0.1% Tween 80, n = 7) or scopolamine bromide (1 mg/kg/0.4 mL/cc, intraperitoneally (*i.p*.; n = 7), once daily for 14 days. Standard behavioral assessments (Y-maze, Novel Object Recognition, Passive Avoidance) were conducted 30 min following changes in cognitive performance (see Supplementary Table S1 for behavioral data). Following testing on Day 14, hippocampal tissues were obtained for all molecular assays, including qPCR, which are described below.

## 2. Results

The D-loop sequences were obtained for seven samples from the control and experimental groups, respectively. In total, we analyzed mitochondrial sequence length of 897 bp per animal. Changes observed in noncoding D-loop region include 9 single-nucleotide polymorphisms (SNPs) and 26 heteroplasmic positions. Identified SNPs were m.15505T>C, m.15751T>G, m.15842G>A, m.16105C>T, m.16171A>T, m.16183C>G, m.16189C>T, m.16202C>A, m.16283G>C. Four out of nine polymorphisms were observed in functional sites mapped by Abhyankar et al. (2009) [22]: central block (CB), and Tfam binding site (TFH) in the D-loop of rats (Table 1). Other SNPs were identified in non-functional regions of D-loop. Rate of observed D-loop point mutations transitions and transversions was comparable (44.4% and 55.6%).

### 2.1. Expression of ahr and gpr35 Genes in Hippocampus

The analyses of gene expression for the *Ahr* and *GPR35* genes was conducted using real-time RT-PCR in the hippocampus from scopolamine and vehicle groups. The results showed that there were no statistically significant differences in the relative quantification (RQ) values between the groups (*p* > 0.05). These findings suggest that the experimental conditions did not induce any notable changes in the transcriptional regulation of these genes. The lack of variation implies that neither *Ahr* nor *GPR35* expression was impacted by the experimental intervention.

### 2.2. Expression of katI, katII, and kmo Genes in Hippocampus

**Figure 1:**
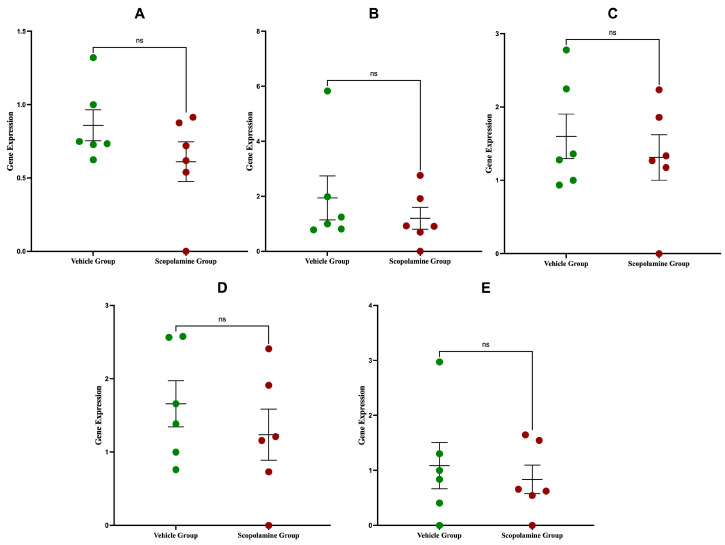
Analyses of gene expression of *ahr* (**A**), *gpr35* (**B**), *katI* (**C**), *katII* (**D**) and *kmo* (**E**) in the experimental (SCOP) group compared to the control (vehicle) group. Data are presented as mean ± SEM, n = 7 animals per group. Statistical significance was determined using Student’s *t*-test or Mann–Whitney test, as appropriate; *p* < 0.05.

The expression levels of *katI*, *katII*, and *kmo* enzymes were similarly evaluated to assess potential changes in KP activity. Across all tested samples, there were no statistically significant differences in gene expression between the scopolamine and vehicle groups (*p* > 0.05). The stability of these enzyme expression levels suggests that the kynurenine metabolic pathway was not significantly affected by the experimental conditions. The results of gene expression analyses for *ahr*, *gpr35*, *katI*, *katII* and *kmo* are shown in Figure 1A–E.

### 2.3. Evaluation of Variability Within Groups

Within each group (scopolamine and vehicle groups), the variability in RQ values was minimal, further emphasizing the robustness of the results. This consistency within groups strengthens the conclusion that no significant transcriptional changes occurred due to the intervention.

## 3. Discussion

In the present study, we analyzed mitochondrial DNA in the D-loop region, which is considered a mutation hotspot and plays a key role in the regulation of mtDNA replication. Our results did not show significant mutations or differences in the level of heteroplasmy between the control and experimental groups. The stability of the D-loop region in the hippocampus of rats after short-term exposure to scopolamine indicates the relative resistance of the mitochondrial genome to acute pharmacological stress induced by scopolamine. The literature emphasizes that mutations in the D-loop region and epigenetic changes such as mtDNA methylation are more often associated with long-term oxidative stress, aging, and chronic neurodegenerative processes [23]. However, our short-term exposure model does not show such lasting changes, which may be due to an effective adaptive response and defense mechanisms, such as increased activity of antioxidant enzymes, e.g., superoxide dismutase (SOD), which neutralizes reactive oxygen species (ROS) and protects mitochondria from damage [24]. The lack of changes in mitochondrial DNA in the hippocampus may be due to the fact that young and healthy organisms are able to maintain mitochondrial homeostasis, even in the face of pharmacological stress induced by scopolamine. It is worth noting that our analyses focused primarily on gene expression and mtDNA stability and did not include direct measurements of mitochondrial function, or enzyme activity related to KP and oxidative stress. Therefore, we cannot rule out the possibility that short-term scopolamine administration could have caused subtle functional changes, invisible at the transcriptomic level. This result is consistent with the literature indicating that mitochondria remain relatively stable in the short term after acute stress, and their dysfunction usually occurs in chronic disease states [25]. In the clinical context, this mitochondrial resistance may represent a natural protective barrier against damage induced by short-term neurochemical perturbations, such as those observed in states of transient memory loss or amnesia. Furthermore, the stability of mtDNA in response to scopolamine suggests that short-term cognitive deficits induced by muscarinic receptor blockade do not result in permanent mitochondrial damage. This may also indicate that short-term therapeutic interventions that induce a similar profile of action should not lead to long-term mitochondrial dysfunction, which is important when assessing the risks and benefits of neuropharmacological therapies.

In our study, we also did not observe significant changes in the expression of *ahr*, *gpr35*, and *katI*, *katII*, and *kmo* genes—key elements of the KP—in the hippocampus of rats exposed to short-term scopolamine compared to the control group. The KP, which regulates tryptophan metabolism and is an important modulator of mitochondrial function, NAD+ production, and neuroprotective mechanisms, is activated under conditions of chronic inflammation, neurodegeneration, and chronic oxidative stress [26]. In this context, the lack of significant activation of genes in this pathway suggests that short-term exposure to scopolamine does not activate this metabolic pathway at the transcriptional level. The mechanism of action of scopolamine involves blocking muscarinic acetylcholine receptors (mAChRs), which causes rapid, transient, and reversible disruptions in cholinergic neurotransmission and neuronal signaling. These disruptions primarily affect cognitive functions such as memory and attention, but do not appear to induce long-term molecular changes such as activation of the KP, which, as mentioned above, is more often associated with chronic inflammation or neurodegenerative diseases [27]. The *Ahr*, which is a transcription factor involved in the response to oxidative stress, regulation of tryptophan metabolism, and mitochondrial function also did not show expression changes in our model [28]. The literature indicates that chronic and long-term activation of the *Ahr* is associated with mitochondrial dysfunction, increased oxidative stress, and the development of inflammation and neurodegenerative diseases. As mentioned, *Ahr* activation affects various cellular pathways that can lead to mitochondrial damage and deterioration of neuronal function, which is important in the pathogenesis of such diseases [29]. The lack of *Ahr* activation in our study is therefore consistent with the assumption that short-term cognitive deficits induced by scopolamine do not involve molecular mechanisms typical of chronic oxidative stress and neurodegeneration. The lack of significant changes in the expression of *ahr*, *gpr35* genes and *katI*, *katII* and *kmo* enzymes in the hippocampus after short-term exposure to scopolamine indicates that the molecular mechanisms responsible for reversible cognitive deficits induced by blockade of muscarinic receptors are not associated with the activation of the KP, which is involved in chronic neurodegenerative and inflammatory processes. This means that short-term memory and cognitive disorders, such as those occurring in the course of transient clinical states, may not lead to permanent metabolic or mitochondrial damage, which favors their reversibility and good prognosis. In addition, the observed stability of *ahr* expression and key components of the KP suggest that therapies aimed at modulating these pathways may be more effective and necessary in cases of chronic neurodegenerative disorders than in acute, transient cognitive deficits. Furthermore, the lack of activation of the KP and *Ahr* in the short-term scopolamine model suggests that cognitive deficits induced by this substance are more related to neurotransmission disorders than to mitochondrial pathology or chronic oxidative stress. This distinction has important clinical implications, especially in the context of diagnosis and treatment of cognitive disorders of various etiologies. In delirium, an acute and often reversible cognitive impairment, studies suggest that neuroinflammatory mechanisms and activation of the kynurenic pathway may play a significant role, especially when triggered by systemic inflammation or infection [30]. However, in many cases, therapies aimed at modulating the kynurenic pathway or using antioxidants have shown limited benefit. Instead, effective treatment often relies on rapid restoration of neurotransmission, particularly via cholinergic pathways. Similarly, cognitive impairments induced by anticholinergic drugs in elderly patients tend to be reversible and are primarily related to temporary blockade of receptors rather than permanent activation of inflammatory pathways or mitochondrial dysfunction [31]. In this regard, avoiding long-term use of anticholinergic drugs and ensuring their rapid withdrawal may promote full recovery of cognitive function. In contrast, neurodegenerative diseases such as Alzheimer’s and Parkinson’s disease are characterized by chronic activation of *Ahr* and the kynurenine pathway, which are associated with progressive mitochondrial dysfunction and persistent oxidative stress. In these conditions, targeting these pathways has therapeutic potential and is currently the subject of extensive clinical trials. Our findings emphasize that short-term, reversible cognitive deficits, such as those induced by scopolamine, should be distinguished from chronic neurodegenerative processes. Clinically, this suggests that in patients experiencing acute cognitive impairments of potentially reversible origin, treatment focused on restoring normal cholinergic neurotransmission may be sufficient without the need for anti-inflammatory or antioxidant interventions. Moreover, the lack of activation of the *Ahr* and KPs in our model may reflect protective and compensatory mechanisms in the young, healthy brain that prevent the progression from transient cognitive impairment to chronic, irreversible states. This highlights the importance of early diagnosis and timely intervention to halt the development of permanent cognitive deficits.

The limitations of the present study include the relatively short duration of exposure, the focus only on the hippocampus, and the limited range of analyzed pathways, which restrict the generalization of results. Future studies should examine longer-term exposure, additional brain regions, broader molecular mechanisms, and investigations in vulnerable groups.

## 4. Materials and Methods

### 4.1. Animals

The experiment was conducted on 14 male Wistar rats, aged 8 weeks, sexually mature, and weighing between 190 and 230 g. The animals originated from a licensed breeding facility at the Experimental Medicine Center of the Medical University of Lublin and were kept under appropriate living conditions throughout the experiment, in accordance with the Regulation of the Minister of Agriculture and Rural Development dated 29 April 2022, on the minimum requirements for facilities and the minimum care standards for animals housed in such centers (Journal of Laws 2022, item 1021 [32]). During the study, the rats were housed two per cage and had continuous, free access to drinking water and nutritious feed free of contaminants. Experimental procedures were performed between 8:00 a.m. and 3:00 p.m. Prior to the experiment, the rats underwent a 7-day habituation procedure aimed at familiarizing the animals with human touch and smell to minimize stress during experimental handling. All animals were randomly assigned to their experimental groups. The final number of animals in each group was determined according to statistical requirements (Student’s *t*-test, ANOVA), as well as compliance with ARRIVE guidelines and the 3R principle. The animal study was approved by the Local Ethical Committee under approval number 12/2022.

### 4.2. Experimental Design

The following chemicals were used in the study: scopolamine hydrobromide (SCOP, S0929) and phosphate-buffered saline (PBS, P2272), both obtained from Sigma-Aldrich (St. Louis, MO, USA). Additionally, Tween 80 (2% polyoxyethylene sorbitan monooleate; POCH, Gliwice, Poland) and sterile 0.9% sodium chloride solution (NaCl) obtained from Sigma-Aldrich (St. Louis, MO, USA) were employed. The vehicle for the solution of the substances administered was prepared daily. Sterile-saline or phosphate-buffered saline with 0.1% Tween 80, a solubilizing agent to improve dissolution, enhance solubility of hydrophobic compounds, and ensure homogeneity of the solution. Scopolamine hydrobromide (SCOP, Sigma-Aldrich, St. Louis, MO, USA) powder was dissolved in the vehicle to a 0.4 mg/mL solution. Tween 80 was used as a solubilizing agent to improve dissolution, ensure homogeneity of the solution, and enhance solubility of hydrophobic compounds. The rats used in the study were randomly assigned to two groups, seven animals in each (n = 7 per group, total n = 14). Group 1 (vehicle control) received sterile 0.9% NaCl solution containing 0.1% Tween 80, which matched the vehicle used for SCOP administration; alternatively, phosphate-buffered saline (PBS) could be used if buffering were required. Group 2 (SCOP) received scopolamine hydrobromide dissolved in the same vehicle. The substances were administered i.p. once daily at the same time for 14 consecutive days. The dose of scopolamine hydrobromide was 1 mg/kg body weight, corresponding to 0.25 mL of solution per 100 g of rat body weight. Each injection was performed manually by gently restraining the animal, and the procedure took no longer than 30 s. All experimental procedures, including administration, behavioral testing, and tissue sampling, were performed according to a fixed schedule: 30 min after the daily administration of SCOP or vehicle, animals underwent behavioral tests, and on day 14, hippocampal tissue was collected for molecular analyses. All molecular assays, including qPCR, were performed on hippocampal tissue obtained from the same seven animals per group (n = 7 biological replicates per group). Data are presented as mean ± SEM.

### 4.3. DNA Extraction and Quality Assessment

Genomic DNA was isolated from the hippocampus tissue using the DNeasy Blood & Tissue Kit (Qiagen, Hilden, Germany) according to the manufacturer’s instructions. The DNA concentration was measured spectrophotometrically using a NanoDrop™ One/OneC Microvolume UV-Vis spectrophotometer (Thermo Fisher Scientific, Waltham, MA, USA), and DNA quality was assessed by electrophoretic separation on a 1.5% agarose gel (multiSUB™ Maxi, Cleaver Scientific Ltd., Rugby, UK) stained with SimplySafe™ (EURx) [33].

### 4.4. PCR Amplification of mtDNA D-Loop Region

The mitochondrial DNA D-loop region was amplified by polymerase chain reaction (PCR) using the primers forward (F) 5′-ATAAACATTACTCTGGTCTTGTAAACC-3′ and reverse (R) 3′-ATTAATTATAAGGCAGGACCAAACCT-5′ [23]. PCR amplification conditions were based on the protocol described by Pagès et al. (2010) and included an initial denaturation at 94 °C for 4 min, followed by 40 cycles of denaturation at 94 °C for 30 s, annealing at 54 °C for 30 s, elongation at 72 °C for 2 min, and a final extension at 72 °C for 10 min [34]. PCR products were visualized on a 1.5% agarose gel stained with SimplySafe™ [33].

### 4.5. Library Preparation and Sequencing

PCR products were purified using Ampure XP magnetic beads (Beckman Coulter, Brea, CA, USA), and their concentrations were measured fluorometrically using a Qubit 3.0 fluorimeter (Thermo Fisher Scientific, Waltham, MA, USA). Appropriately diluted PCR products were used for library preparation. Approximately 1 ng of PCR DNA template was used as input for shotgun library construction using the Nextera XT Kit (Illumina, San Diego, CA, USA), following the manufacturer’s protocol. Libraries were sequenced on an Illumina MiSeq sequencer using a 600-cycle kit (v3) in paired-end mode targeting a minimum coverage of 100×. Obtained mitochondrial DNA sequences were compared with the NCBI reference sequence (NC_001665.2) using the Unipro UGENE bioinformatics software version 34.0 [35].

### 4.6. RNA Isolation and cDNA Synthesis

For gene expression analyses, cellular RNA was isolated from hippocampus homogenate supernatants using RNeasy Mini QIAcube Kit (Qiagen, Germany) and subsequently processed on the QiaCube automated workstation with an RNA isolation protocol. The concentration and purity of the isolated RNA were determined using a Nanodrop Lite spectrophotometer (Thermo Fisher Scientific, Waltham, MA, USA). Reverse transcription of RNA to complementary DNA (cDNA) was performed using a Veriti thermocycler (Thermo Fisher Scientific, Waltham, MA, USA) and the High-Capacity cDNA Reverse Transcription Kit (Thermo Fisher Scientific, Waltham, MA, USA). Each reaction contained 1 µg of RNA in 10 µL water mixed with 10 µL of the reverse transcription master mix, comprising 2 µL of 10× RT buffer, 0.8 µL of 25× dNTP mix (100 mM), 2 µL of 10× RT random primers, 1 µL of RNase inhibitor (20 U/µL), 1 µL of reverse transcriptase (50 U/µL), and 3.2 µL of ultrapure water. Samples were mixed, briefly centrifuged, and subjected to the following thermal program: 25 °C for 10 min, 37 °C for 120 min, 85 °C for 5 min, and then held at 4 °C until further use.

### 4.7. Quantitative Real-Time PCR (qRT-PCR)

Quantitative real-time PCR (qRT-PCR) was conducted to assess gene expression levels using 96-well plates on a QuantStudio™ 12K Flex system (Thermo Fisher Scientific, Waltham, MA, USA). Each reaction had a total volume of 25 µL, comprising 1 µL cDNA, 9.25 µL of water, 1.25 µL of target-specific probe, and 12.5 µL of Master Mix buffer (Thermo Fisher Scientific, Waltham, MA, USA). TaqMan probes targeting the following genes were used: *katI* (ccbl1, Mm00549584_m1), *katII* (aadat, Mm00496169_m1), *ahr* (Rn00565750_m1), *gpr35* (Rn02748664_s1), *kmo* (Rn00665313_m1) as well as the housekeeping gene actb (beta-actin, Rn00667869_m1). Amplifications were carried out for 40 cycles. Six technical replicates were performed for each sample. Gene expression data were analyzed using Expression Suite software ver. 1.2.2 (Thermo Fisher Scientific, Waltham, MA, USA), and relative quantification (RQ) values normalized to the control group were used for statistical analysis.

### 4.8. Statistical Analysis

Statistical analysis was performed using GraphPad Prism 10 (GraphPad Software, San Diego, CA, USA). Before selecting the statistical test, the distribution of data was assessed using the Shapiro–Wilk test. For data fulfilling the assumption of normal distribution, Student’s *t*-test was used for comparisons between two independent groups –control and experimental group. For data that did not meet the criteria for normal distribution, the nonparametric Mann–Whitney test was used. All *p*-Values were presented as two-tailed, and the level of statistical significance was set at *p* < 0.05. Data are presented as mean ± SEM, with n = 7 animals per group for all analyses, including qPCR experiments.

## 5. Conclusions

The present study shows that short-term exposure to scopolamine does not induce significant mutations or heteroplasmy changes in the D-loop region of mtDNA in the rat hippocampus, indicating the stability of the mitochondrial genome under acute pharmacological stress. No significant changes in the expression of *katI*, *katII*, *kmo* and *ahr* and *gpr35* genes were observed, suggesting a lack of activation of this pathway in transient cognitive deficits induced by muscarinic receptor blockade. These results emphasize the difference between reversible cognitive deficits and chronic neurodegenerative processes, in which the KP and mtDNA damage play a key role. The stability of mtDNA and the lack of activation of *Ahr* and KP suggest that short-term cognitive impairments induced by scopolamine are more related to neurotransmission disorders than to chronic oxidative stress or mitochondrial dysfunction. These observations have clinical and therapeutic implications, indicating that in acute, reversible cognitive impairment, treatment should focus on restoring normal neurotransmission rather than on antioxidant or anti-inflammatory interventions.

## Figures and Tables

**Table 1:** mtDNA variants observed in functional sites in the D-loop of rats. No significant differences in the number or type of single-nucleotide polymorphisms (SNPs) were observed between control rats and those exposed to short-term scopolamine. No differences in SNP number or type were observed between groups.

Locus	Description	Range	Number of Variants	mtDNA Variant
ETAS1	Termination-associated sequence	15,446–15,503	0	
TAS-D		15,497–15,511	1	m.15505T>C
TAS-C		15,520–15,531	0	
TAS-B		15,541–15,554	0	
TAS-A		15,571–15,584	0	
ETAS2		15,511–15,572	0	
CB	Central Block	15,673–15,979	2	m.15751T>G
				m.15842G>A
CSB1	Conserved sequence block 1	16,027–16,052	0	
CSB2	Conserved sequence block 2	16,083–16,099	0	
CSB3	Conserved sequence block 3	16,116–16,133	0	
TFL	Tfam binding site	16,212–16,226	0	
TFH	Tfam binding site	16,267–16,286	1	m.16283G>C

## Data Availability

The data that support the findings of this study are available from the corresponding author upon reasonable request. The data are not publicly available due to ethical restrictions and because further analyses are ongoing.

## Supplementary Materials

The following supporting information can be downloaded at https://www.mdpi.com/article/10.3390/ijms26209883/s1.

## Abbreviations

The following abbreviations are used in this manuscript:

*Ahr*	Aryl hydrocarbon receptor
ATP	Adenosine triphosphate
CB	Central block
F	Forward primer
*GPR35*	G protein-coupled receptor 35
*KAT I*	Kynurenine aminotransferase I
*KAT II*	Kynurenine aminotransferase II
*KMO*	Kynurenine 3-monooxygenase
KP	The Kynurenine Pathway
KYNA	Kynurenic acid
mtDNA	Mitochondrial DNA
PCR	Polymerase chain reaction
qRT-PCR	Quantitative real-time PCR
R	Reverse primer
ROS	Reactive oxygen species
RQ	Relative quantification
SNPs	Single-nucleotide polymorphisms
SOD	Superoxide dismutase
TFAM	Mitochondrial transcription factor A
TFH	Tfam binding site
