# Supplementary material for: Mitochondrial Integrity and Kynurenine Pathway Enzyme Dynamics in the Hippocampus of Rats with Scopolamine-Induced Cognitive Deficits
Source: Int J Mol Sci. 2025 Oct 11;26(20):9883. doi: 10.3390/ijms26209883 (PMC12563731; doi:10.3390/ijms26209883)
Supplement: Supplementary file 1 [file ijms-26-09883-s001.zip › ijms-3817532-supplementary.pdf]

Supplementary Table S1

**Experimental design and behavioral testing schedule**				
**Group**	**Treatment (daily, 14 days)**	**Behavioral testing (30 min post-administration)**	**Days when differences were noted**	**Tissue collection**
Vehicle control	0.9% NaCl + 0.1% Tween 80 (*i.p.)*	Y-maze (day 8), NOR (days 9–11), PA (days 12–14)*	–	Day 14 (hippocampus)
Scopolamine (SCOP)	SCOP 1 mg/kg (*i.p.*)	Y-maze (day 8), NOR (days 9–11), PA (days 12–14)*	Day 8 (Y-maze); Days 13–14 (PA)	Day 14 (hippocampus)
